# Inhibition of cyclooxygenase-1 by nonsteroidal anti-inflammatory drugs demethylates MeR2 enhancer and promotes *Mbnl1* transcription in myogenic cells

**DOI:** 10.1038/s41598-020-59517-y

**Published:** 2020-02-13

**Authors:** Kun Huang, Akio Masuda, Guiying Chen, Samira Bushra, Masayoshi Kamon, Toshiyuki Araki, Masanobu Kinoshita, Bisei Ohkawara, Mikako Ito, Kinji Ohno

**Affiliations:** 1 Division of Neurogenetics, Center for Neurological Diseases and Cancer, Nagoya University Graduate School of Medicine, Nagoya, Aichi Japan; 2 Department of Peripheral Nervous System Research, National Institute of Neuroscience, National Center of Neurology and Psychiatry, Kodaira, Tokyo Japan; 3 Mumin Clinic Omiyakita, Saitama, Saitama, Japan

**Keywords:** Gene expression, Neuromuscular disease

## Abstract

Muscleblind-like 1 (MBNL1) is a ubiquitously expressed RNA-binding protein, which is highly expressed in skeletal muscle. Abnormally expanded CUG-repeats in the *DMPK* gene cause myotonic dystrophy type 1 (DM1) by sequestration of MBNL1 to nuclear RNA foci and by upregulation of another RNA-binding protein, CUG-binding protein 1 (CUGBP1). We previously reported that a nonsteroidal anti-inflammatory drug (NSAID), phenylbutazone, upregulates MBNL1 expression in DM1 mouse model by demethylation of MeR2, an enhancer element in *Mbnl1* intron 1. NSAIDs inhibit cyclooxygenase (COX), which is comprised of COX-1 and COX-2 isoforms. In this study, we screened 29 NSAIDs in C2C12 myoblasts, and found that 13 NSAIDs enhanced *Mbnl1* expression, where COX-1-selective NSAIDs upregulated *Mbnl1* more than COX-2-selective NSAIDs. Consistently, knockdown of COX-1, but not of COX-2, upregulated MBNL1 expression in C2C12 myoblasts and myotubes, as well as in myotubes differentiated from DM1 patient-derived induced pluripotent stem cells (iPSCs). Luciferase assay showed that COX-1-knockdown augmented the MeR2 enhancer activity. Furthermore, bisulfite sequencing analysis demonstrated that COX-1-knockdown suppressed methylation of MeR2. These results suggest that COX-1 inhibition upregulates *Mbnl1* transcription through demethylation of the MeR2 enhancer. Taken together, our study provides new insights into the transcriptional regulation of *Mbnl1* by the COX-1-mediated pathway.

## Introduction

Muscleblind-like (MBNL) is a multifunctional RNA binding protein that modulates diverse RNA metabolisms, including splicing, polyadenylation, stability, and localization of mRNA^1–3^. There are three isoforms of MBNL in mammals (MBNL1, MBNL2, and MBNL3), in which MBNL1 is the most ubiquitously expressed isoform and is highly expressed in skeletal muscle^4^. Expression of MBNL1 is elevated in skeletal muscle during myogenic differentiation^5^. Knockout of *Mbnl1* results in pathology reminiscent of myotonic dystrophy type 1 (DM1) with abnormal terminal muscle differentiation^6–8^.

DM1 is the most common form of muscular dystrophies in adults, and is caused by abnormal expansion of CTG repeats in the 3′ untranslated region of the myotonic dystrophy protein kinase (*DMPK*) gene on chromosome 19 ^9,10^. The transcribed CUG repeats from the CTG repeats make abnormal RNA foci in the nucleus, leading to sequestration of MBNL1 and upregulation of another RNA binding protein, CUG-binding protein 1 (CUGBP1)^11^. Downregulation of MBNL1 availability leads to aberrant regulation of alternative splicing of hundreds of genes, which causes various manifestations such as myotonia, progressive muscle wasting, cataracts, insulin resistance, cardiac arrhythmia, and intellectual deficits^8,12,13^.

Several therapeutic strategies for DM1 are currently under investigation^14–16^. Induction of MBNL1 expression is one of the promising therapies for DM1. Overexpression of *Mbnl1* in skeletal muscle with an adeno-associated viral vector rescues disease-associated muscle hyperexcitability or myotonia in a transgenic mouse model for DM1^17,18^. Similarly, overexpression of human *MBNL1* alleviated muscle degeneration in a fly model of DM1^19^. In *Drosophila*, *Mbnl*, the homolog of human MBNL1, harbors two enhancers located in intron 2, which drive specific gene expression in embryonic somatic muscle and in the central nervous system^20^. Enhancers homologous to the *Drosophila* enhancers in mammalian *MBNL1* were not known. We previously identified an enhancer for *Mbnl1* transcription, designated MeR2 (the methylated region 2), in intron 1 of mouse *Mbnl1*^21^. Our analysis revealed that demethylation of MeR2 enhances *Mbnl1* expression in C2C12 myoblasts as well as in mouse skeletal muscle^21^.

Nonsteroidal anti-inflammatory drugs (NSAIDs) are widely used for inflammatory diseases including neurodegenerative and neuromuscular diseases^22–24^. NSAIDs inhibit cyclooxygenase (COX) that catalyzes the conversion of arachidonic acid to prostanoids. There are two COX isoforms: COX-1 is constitutively expressed in most tissues, and COX-2 is inducibly expressed in response to inflammation^25–27^. Selective inhibitors for each COX isoform have been developed, although most classical NSAIDs inhibit both COX isoforms^28^. We^21^ and others^29^ reported beneficial effects of NSAIDs on DM1. Screening of 400 preapproved drugs using a *Drosophila* model for DM1 disclosed 10 candidate drugs including two NSAIDs (ketoprofen and indomethacin)^29^. We previously reported that phenylbutazone, a non-selective NSAID, ameliorates muscle weakness and muscle pathology by enhancement of MBNL1 expression, as well as by inhibition of the interaction between MBNL1 and CUG RNA^21^. Phenylbutazone augments transcription of *Mbnl1* mRNA by demethylating the MeR2 enhancer.

In the current study, we screened 29 NSAIDs for upregulation of *Mbnl1* in C2C12 mouse myoblasts, and found that COX-1-selective NSAIDs were more effective than COX-2-selective NSAIDs. Knockdown of COX-1, but not of COX-2, enhanced demethylation of the MeR2 enhancer and increased *Mbnl1* transcription. We show that *Mbnl1* transcription is suppressed by COX-1-mediated pathway, and propose that COX-1-selective NSAIDs are potential drugs for DM1 patients.

## Results

### Drug screening for 29 NSAIDs to elevate *Mbnl1* mRNA expression

**Figure 1:**
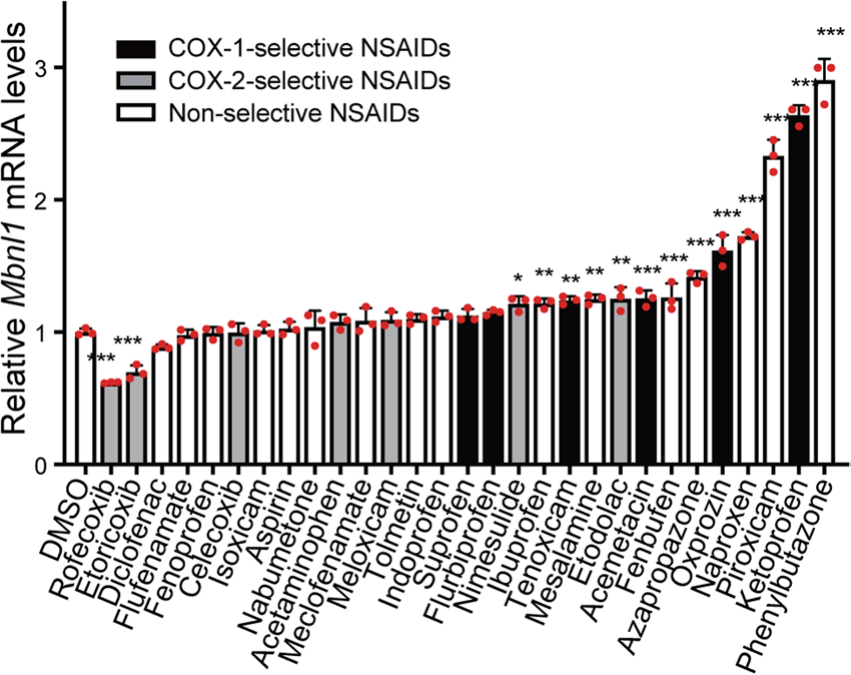
Screening of 29 NSAIDs on *Mbnl1* mRNA expression. Undifferentiated C2C12 cells were cultured with 100 μM each of NSAIDs for 24 h. Expression levels of *Mbnl1* and *Gapdh* were quantified by real-time RT-PCR. Expression level of *Mbnl1* was normalized to that of *Gapdh*, and also to control cells with only DMSO. Mean and SD (*n* = 3 culture dishes) are indicated with individual values in red dots. *p < 0.05, **p < 0.01, and ***p < 0.001 compared to DMSO alone by Student’s *t*-test with Bonferroni multiple comparison correction.

We previously reported that phenylbutazone, a non-selective NSAID, enhances MBNL1 expression in myogenic cells^21^. We here screened 29 NSAIDs to examine a class effect of NSAIDs on enhancement of *Mbnl1* expression. C2C12 myoblasts were incubated for 24 h with 100 μM each NSAID, followed by quantification of *Mbnl1* mRNA using real-time RT-PCR. Our screening identified 13 drugs that increased *Mbnl1* mRNA, in which phenylbutazone had the largest effect (Fig. 1). The second-ranked drug, ketoprofen, was previously reported to suppress CUG-induced lethality in *Drosophila*^20^. We also confirmed that the fourth-ranked drug, naproxen, elevated mRNA and protein levels of MBNL1 in C2C12 cells (Supplementary Fig. S1b,c). Four out of six COX-1-selective NSAIDs and two out of seven COX-2-selective NSAIDs increased *Mbnl1* mRNA (Fig. 1). Wilcoxon rank sum test showed that COX-1-selective NSAIDs increased *Mbnl1* mRNA more than COX-2-selective NSAIDs (*p* = 0.022). Additionally, two COX-2-selective NSAIDs, but no COX-1-selective NSAID, downregulated *Mbnl1* mRNA. We also examined the effect of each NSAID at a lower concentration (10 μM) on *Mbnl1* expression in C2C12 cells. We observed that the 6 top-ranked NSAIDs at 100 µM that upregulated *Mbnl1* mRNA more than 1.4-folds were again the 6 top-ranked NSAIDs at 10 μM (red letters in Supplementary Fig. S1a). In addition, at 10 µM, a statistically significant increase of *Mbnl1* mRNA was observed in two COX-1-selective NSAIDs, but in no COX-2-selective NSAID (red letters in Supplementary Fig. S1a). In contrast, at 10 µM, a statistically significant decrease of *Mbnl1* mRNA was observed in no COX-1-selective NSAID, but in two COX-2-selective NSAIDs (blue letters in Supplementary Fig. S1a). Taken together, our analysis suggests that a substantial number of NSAIDs can increase MBNL1 expression, in which COX-1-mediated pathway, rather than COX-2-mediated pathway, plays a pivotal role.

### Knockdown of COX-1, but not of COX-2, upregulates MBNL1 expression in myogenic cells

**Figure 2:**
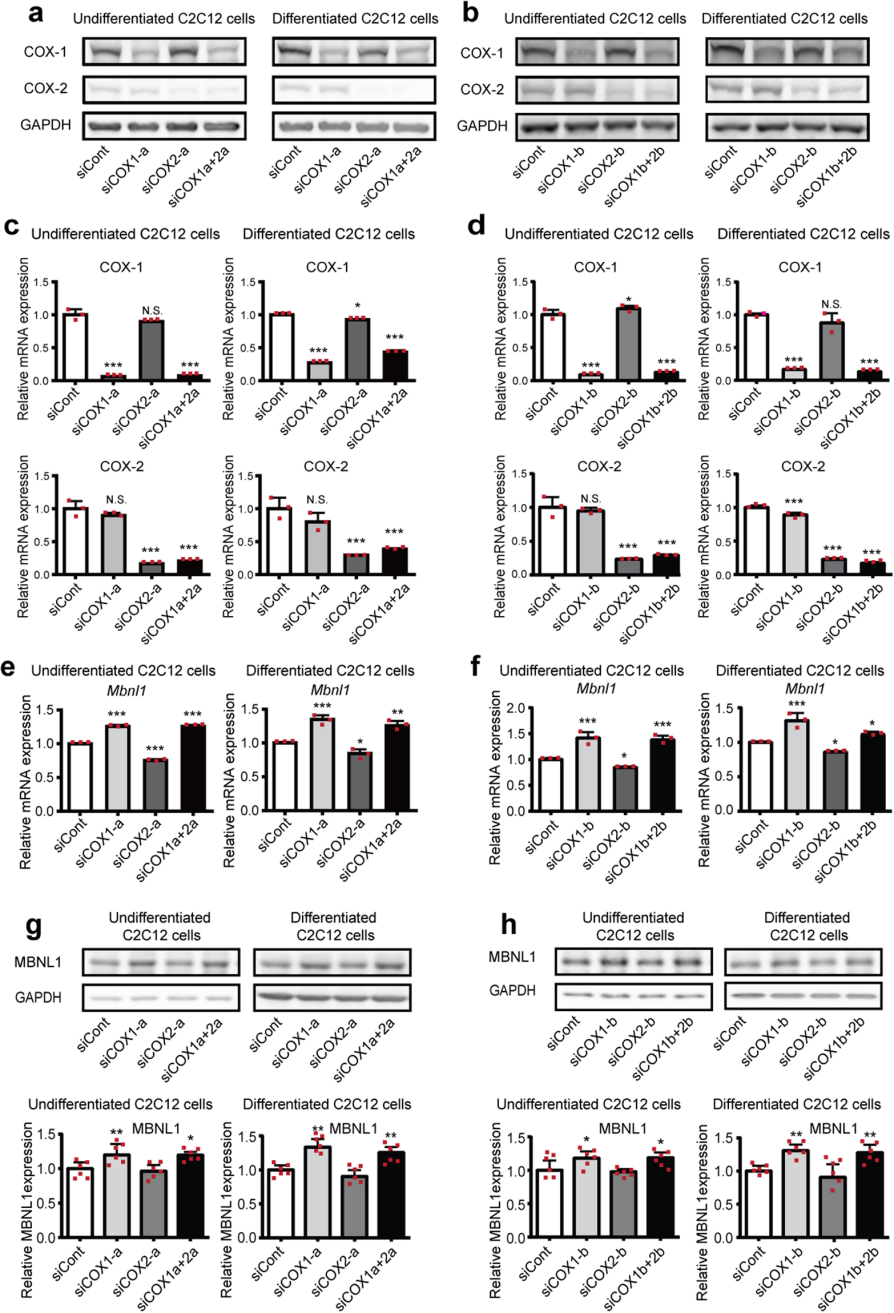
The effect of knockdown of COX-1 and COX-2 on *Mbnl1* mRNA and MBNL1 protein expression in C2C12 cells. Western blotting (**a**,**b**,**g**,**h**) and real-time RT-PCR analysis (**c**–**f**) were performed using undifferentiated C2C12 cells or C2C12 cells differentiated for 5 days. The cells were treated with siRNA against COX-1 (siCOX1-a or siCOX1-b), COX-2 (siCOX2-a or siCOX2-b), both COX-1 and COX-2 (siCOX1a + 2a or siCOX1b + 2b), or control siRNA (siCont). Undifferentiated C2C12 cells were examined 2 days (mRNA levels) or 3 days (protein levels) after the transfection. Differentiated C2C12 cells were examined on day 5 of differentiation. Expression levels of COX-1, COX-2, *Mbnl1* mRNA and MBNL1 protein are normalized to those of *Gapdh* and GAPDH, respectively, and also to siCont-treated cells. Mean and SD (*n* = 3 and 6 culture dishes for real-time RT-PCR and Western blotting, respectively) are indicated with individual values in red dots. *p < 0.05, **p < 0.01 and ***p < 0.001 by Student’s *t*-test with Bonferroni multiple comparison correction.

To dissect the regulation of MBNL1 expression through COX-1-mediated and COX-2-mediated pathways, we knocked down COX-1 and COX-2, and examined *Mbnl1* mRNA levels and MBNL1 protein levels in C2C12 cells. We first confirmed that our siRNAs efficiently knocked down COX-1 and COX-2 (Fig. 2a–d). We found that knockdown of COX-1 with two independent siRNAs (siCOX1-a and -b) upregulated expression of *Mbnl1* mRNA approximately 1.3-folds while knockdown of COX-2 downregulated *Mbnl1* mRNA in C2C12 myoblasts. In addition, double knockdown of COX-1 and COX-2 increased *Mbnl1* mRNA in these cells (Fig. 2e,f). Western blotting analysis also showed elevation of MBNL1 protein expression in COX-1-knocked down cells (Fig. 2g,h). Since the expression level of MBNL1 is upregulated in differentiated myogenic cells^5,30,31^, we further analyzed the *Mbnl1* mRNA and MBNL1 protein levels in differentiated C2C12 myotubes. Similar to the results of undifferentiated C2C12 myoblasts, knockdown of COX-1 increased *Mbnl1* mRNA and MBNL1 protein in differentiated C2C12 myotubes (Fig. 2e–h).

**Figure 3:**
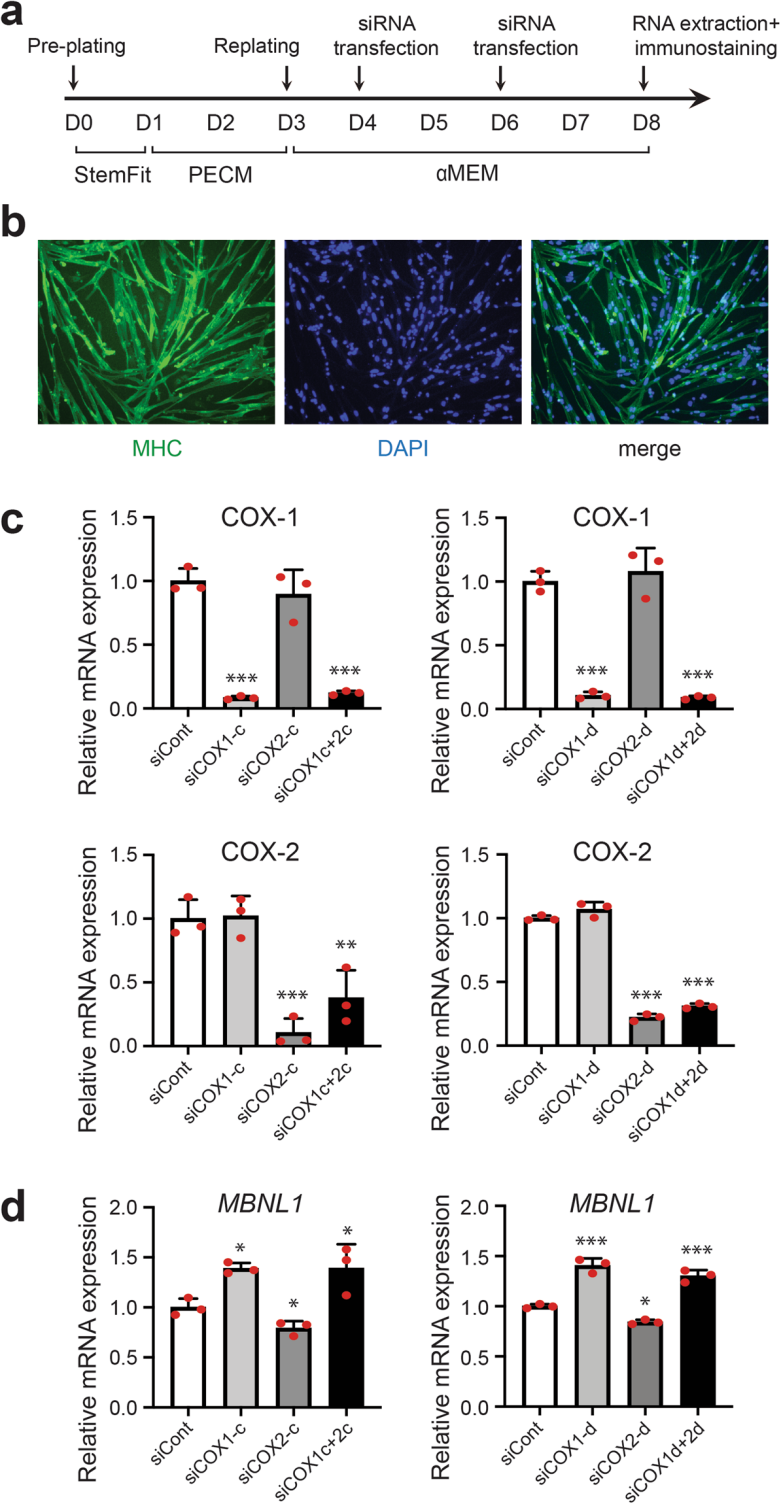
The effect of knockdown of COX-1 and COX-2 on *MBNL1* mRNA expression in myotubes differentiated from MyoD-DM1-iPSCs. (**a**) A schematic diagram of the differentiation of MyoD-DM1-iPSCs and the transfection of siRNAs. siRNAs against COX-1 (siCOX1-c or siCOX1-d), COX-2 (siCOX2-c or siCOX2-d), both COX-1 and COX-2 (siCOX1c + 2c or siCOX1d + 2d), or control siRNA (siCont) were introduced at differentiation day 4 and again at differentiation day 6. (**b**) Immunofluorescence images of myosin heavy chain (MHC) staining of myotubes differentiated from MyoD-DM1-iPSCs on differentiation day 8. (**c**,**d**) Real-time RT-PCR analysis was performed using myotubes differentiated from MyoD-DM1-iPSCs on differentiation day 8. Expression levels of *Ptgs1* (COX-1) mRNA and *Ptgs2* (COX-2) mRNA (**c**), and *MBNL1* mRNA (**d**) are normalized to that of *GAPDH* and also to siCont-treated cells. Mean and SD (*n* = 3 culture dishes) are indicated with individual values in red dots. *p < 0.05, **p < 0.01 and ***p < 0.001 by Student’s *t*-test with Bonferroni multiple comparison correction.

We next examined the effect of knockdown of COX-1 and COX-2 on MBNL1 expression in DM1 patient-derived induced pluripotent stem cells (MyoD-DM1-iPSCs), which were differentiated into myotubes by the induction of MyoD expression (Fig. 3a,b)^32,33^. Similar to C2C12 myoblasts and myotubes (Fig. 2e,f), knockdown of COX-1 significantly upregulated expression of *MBNL1* mRNA, while knockdown of COX-2 downregulated *MBNL1* mRNA in myotubes differentiated from MyoD-DM1-iPSCs. Double knockdown of COX-1 and COX-2 increased *MBNL1* mRNA in these cells (Fig. 3c,d). The upregulation of *Mbnl1* mRNA by COX-1-knockdown was also observed in primary mouse myoblasts and KD3 human myoblast cells (Supplementary Figs. S2 and S3). Taken together, our analysis suggests that MBNL1 expression is suppressed through the COX-1-mediated pathway in myogenic cells.

### The MeR2 enhancer in *Mbnl1* is suppressed through the COX-1-mediated pathway

**Figure 4:**
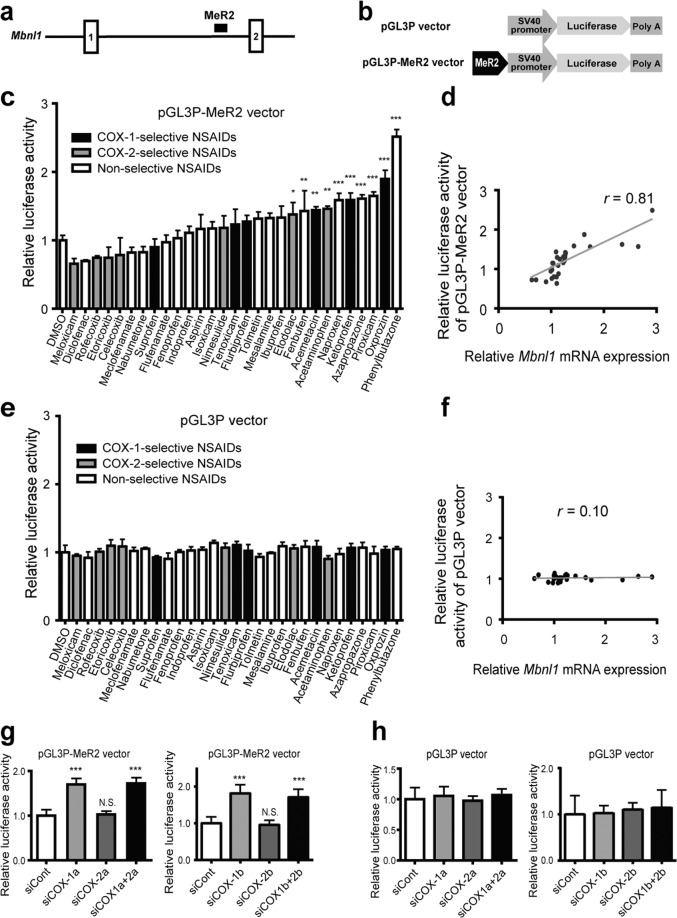
Inhibition of COX-1-mediated pathway activates the MeR2 enhancer in *Mbnl1* intron 1. (**a**) Schematic diagram showing the position of mouse MeR2 enhancer in *Mbnl1* gene. Exons are shown by boxes, introns by thin lines, and MeR2 by a black closed square. (**b**) Schematic diagram of pGL3P vector and pGL3P-MeR2 vector. In pGL3P-MeR2, the MeR2 enhancer region was cloned into pGL3P upstream of the SV40 promoter and the firefly luciferase gene. (**c**,**e**) Luciferase activity of pGL3P-MeR2 (**c**) or pGL3P (**e**) in C2C12 cells treated with NSAIDs. The indicated NSAIDs were added to the culture medium at 24 h after transfection of the luciferase vectors, and were incubated for an additional 24 h. Then, the luciferase activity of pGL3P-MeR2 (**c**) or pGL3P (**e**) was measured, and was normalized to the Renilla luciferase activity of co-transfected pRL-CMV. The ratio was also normalized to that of control cells treated with 0.1% DMSO alone. Mean and SD (*n* = 3 culture dishes) are indicated. (**d**,**f**) Correlation between the relative *Mbnl1* mRNA expression (Fig. 1) and the relative luciferase activity of pGL3P-MeR2 (**d**) or pGL3P (**f**) for each NSAID normalized for 0.1% DMSO. Pearson’s correlation coefficient (*r*) is indicated. (**g**,**h**) Luciferase activity of pGL3P-MeR2 (**g**) or pGL3P (**h**) in COX-1 or COX-2-knocked down cells. C2C12 cells were transfected with siRNAs against COX-1 (siCOX1-a or siCOX1-b), COX-2 (siCOX2-a or siCOX2-b), both COX-1 and COX-2 (siCOX1a + 2a or siCOX1b + 2b), or control siRNA (siCont) at 24 h after transfection of the luciferase vectors, and were incubated for an additional 48 h. Firefly luciferase activity was normalized to the Renilla luciferase activity of co-transfected pRL-CMV, and also to siCont-transfected cells. Mean and SD (*n* = 8 culture dishes) are indicated. *p < 0.05, **p < 0.01, ***p < 0.001, and N.S., not significant by Student’s *t*-test with Bonferroni multiple comparison correction.

We previously identified a transcription enhancer, named MeR2, in *Mbnl1* intron 1, and reported that the demethylation of the MeR2 enhancer upregulated *Mbnl1* expression (Fig. 4a)^21^. To examine whether NSAIDs upregulate the activity of the MeR2 enhancer, we inserted the MeR2 enhancer upstream of the SV40 promoter and the firefly luciferase cDNA to make pGL3P-MeR2 (Fig. 4b). C2C12 myoblasts were introduced with pGL3P-MeR2 or pGL3P, and were treated with one of the 29 NSAIDs at 100 μM for 24 h. Luciferase assay revealed that 10 out of the 29 NSAIDs significantly increased the luciferase activity of pGL3P-MeR2 (Fig. 4c), while no drugs affected that of pGL3P (Fig. 4e). Furthermore, fold inductions of *Mbnl1* mRNA expression by NSAIDs (Fig. 1) were correlated well with those of luciferase activity of pGL3P-MeR2 (Pearson’s correlation coefficient, *r* = 0.81, *p* < 0.001, Fig. 4d), but not those of pGL3P vector (Pearson’s correlation coefficient, *r* = 0.10, *p* = 0.61, Fig. 4f). We also examined whether the COX-1-mediated pathway is specifically involved in the activity of the MeR2 enhancer. As predicted, knockdown of COX-1, but not of COX-2, upregulated the luciferase activity of pGL3P-MeR2 (Fig. 4g,h). Overall, our results suggest that the MeR2 enhancer is suppressed through the COX-1-mediated pathway.

**Figure 5:**
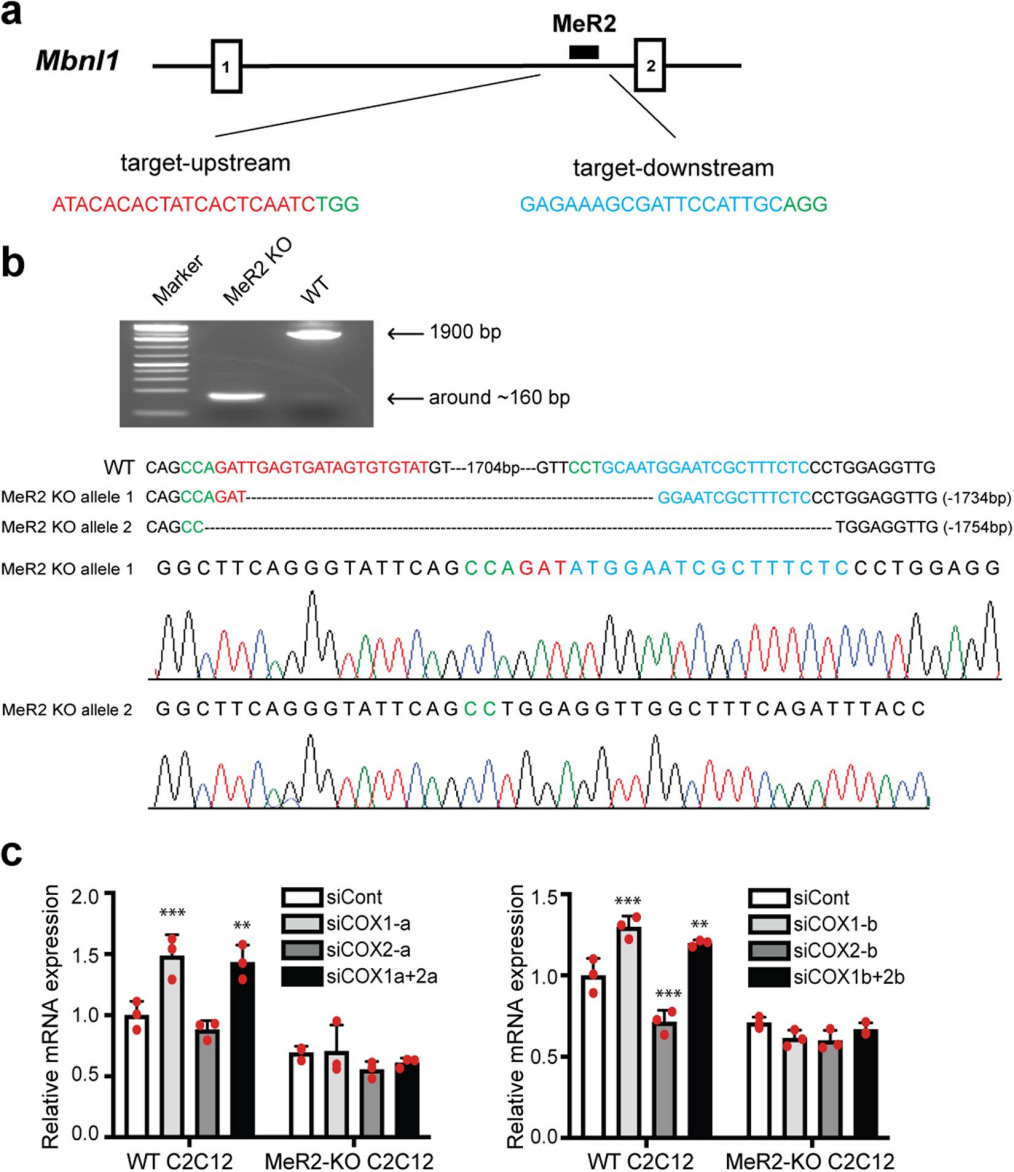
Deletion of the MeR2 enhancer by CRISPR/Cas9 system compromises the effect of COX-1 on *Mbnl1* expression. The MeR2 enhancer in C2C12 myoblasts was knocked out (KO) using CRISPR/Cas9 system. (**a**) Schematic diagram of the target location and sequences of the two sgRNAs (sgRNA-1 and sgRNA-2) designed to delete MeR2. The protospacer-adjacent motifs (PAM) are marked in green and the target sequences of the sgRNAs are in red and blue, respectively. (**b**) PCR analysis and Sanger sequencing analysis showing the deletion of MeR2 in MeR2-KO C2C12 cells. The sequence of the non-edited cells (WT) is shown above. (**c**) The effect of knockdown of COX-1 and COX-2 on *Mbnl1* mRNA expression in WT C2C12 cells and MeR2-KO C2C12 cells. The cells were treated with siRNA against COX-1 (siCOX1-a or siCOX1-b), COX-2 (siCOX2-a or siCOX2-b), both COX-1 and COX-2 (siCOX1a + 2a or siCOX1b + 2b), or control siRNA (siCont). Real-time RT-PCR analysis was performed 48 h after transfection. Expression levels of *Mbnl1* mRNA are normalized to that of *Gapdh*, and also to the siCont-treated WT C2C12 cells. Mean and SD (*n* = 3 culture dishes) are indicated with individual values in red dots. **p < 0.01 and ***p < 0.001 com*p*ared to siCont by Student’s *t*-test with Bonferroni multiple comparison correction.

To investigate the role of the MeR2 enhancer more directly, we deleted the genomic MeR2 region in C2C12 myoblasts by CRISPR/Cas9 system using two single-guide RNAs (sgRNAs) targeting the upstream and downstream sites of MeR2, respectively (Fig. 5a). We obtained one MeR2-knockout C2C12 cell line (MeR2-KO C2C12), where MeR2 was deleted in both alleles (Fig. 5b). We confirmed that knockdown of COX-1 with two independent siRNAs (siCOX1-a and -b) had no effect on *Mbnl1* mRNA expression in MeR2-KO C2C12, but upregulated *Mbnl1* mRNA expression in the control cell line (WT C2C12), which retained MeR2 (Fig. 5c). Thus, COX-1 requires the MeR2 enhancer to suppress *Mbnl1* mRNA expression.

### The MeR2 enhancer in *Mbnl1* is methylated through COX-1-mediated pathway

**Figure 6:**
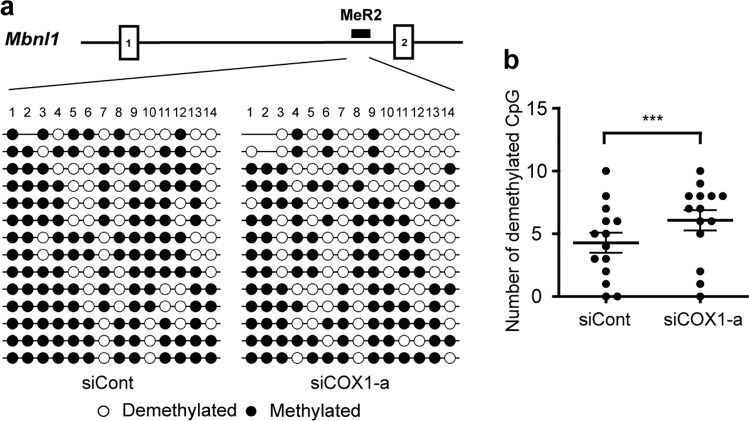
Knockdown of COX-1 suppresses methylation of CpG dinucleotides in MeR2 enhancer region. (**a**) Methylation patterns of the MeR2. C2C12 cells were transfected with the siRNA against COX-1 (siCOX1-a) or control siRNA (siCont), and DNA was extracted on differentiation day 3. Then the samples were treated with bisulfite sodium and cloned into the TA cloning vector for sequencing. For methylation analysis, 14 independent clones were sequenced for each group, and methylations of 14 CpG dinucleotides in MeR2 were analyzed. Methylated and unmethylated CpG sites are shown in closed and open circles, respectively. (**b**) The number of demethylated CpG in MeR2 was counted individually at positions 1 to 14, and is plotted. The mean and SEM are indicated. ***p < 0.001 by paired Student’s *t*-test.

We further analyzed COX-1-dependent regulation of MeR2-methylation in C2C12 myoblasts using bisulfite sequencing. We transfected C2C12 cells with siRNAs against COX-1 or COX-2 on myogenic differentiation day 0, and performed bisulfite sequencing analysis of MeR2 on differentiation day 3. Our analysis revealed that knockdown of COX-1 (Fig. 6), but not of COX-2 (Supplementary Fig. S4), significantly suppressed methylation of MeR2 in C2C12 cells. These results suggest that COX-1 inhibition suppresses methylation of MeR2 and enhances *Mbnl1* transcription in C2C12 myoblasts.

## Discussion

In the current study, we screened 29 NSAIDs for upregulation of MBNL1 using C2C12 mouse myoblasts, and found that 13 NSAIDs significantly enhanced expression of *Mbnl1* mRNA (Fig. 1). COX-1-selective NSAIDs upregulated *Mbnl1* mRNA more than COX-2-selective NSAIDs (Fig. 1). Consistently, knockdown of COX-1, but not of COX-2, upregulated MBNL1 expression in mouse C2C12 myoblasts/myotubes, human KD3 myoblasts/myotubes, mouse primary myoblasts, and myotubes differentiated from DM1 patient-derived iPSCs (Figs. 2 and 3). We also found that knockdown of COX-1 activated the MeR2 enhancer in *Mbnl1* intron 1, and upregulated *Mbnl1* transcription through demethylation of MeR2 (Figs. 4–6). These results suggest that inhibition of the COX-1-mediated pathway upregulates *Mbnl1* mRNA through demethylation of the MeR2 enhancer. Our study disclosed a new role of COX-1 and NSAIDs in MBNL1 expression.

**Figure 7:**
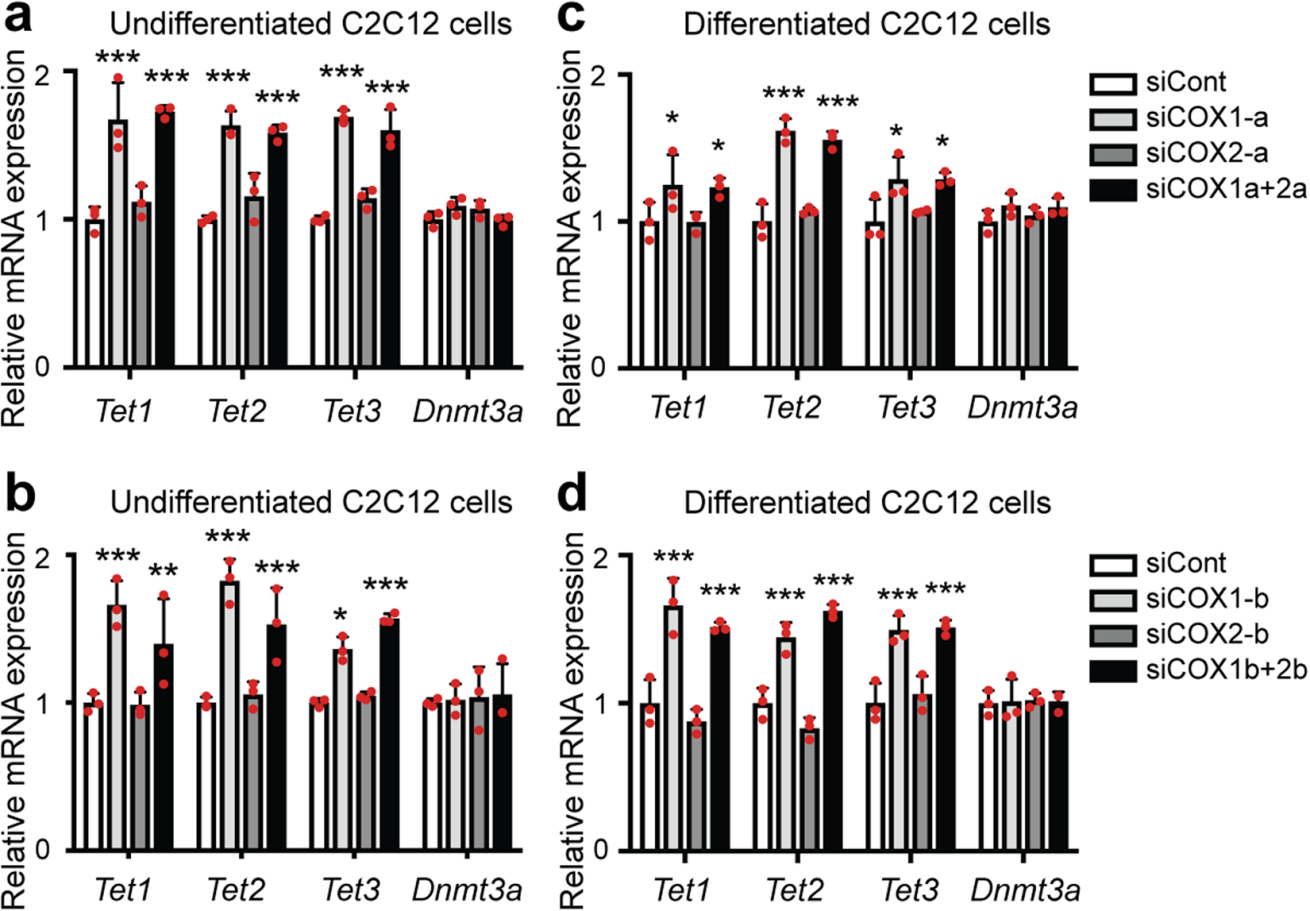
The effects of knockdown of COX-1 or COX-2 on *Tet1*, *Tet2*, *Tet3* and *Dnmt3a* mRNA expression in C2C12 cells. Real-time RT-PCR analysis was performed using undifferentiated C2C12 cells (**a**,**b**) or C2C12 cells differentiated for 5 days (**c**,**d**). The cells were treated with siRNA against COX-1 (siCOX1-a or siCOX1-b), COX-2 (siCOX2-a or siCOX2-b), both COX-1 and COX-2 (siCOX1a + 2a or siCOX1b + 2b), or control siRNA (siCont). Undifferentiated C2C12 cells were examined on 2 days after the transfection. Differentiated C2C12 cells were examined on differentiation day 5. Expression levels of *Tet1*, *Tet2*, *Tet3*, and *Dnmt3a* mRNA are normalized to that of *Gapdh* and also to siCont-treated cells. Mean and SD (*n* = 3 culture dishes) are indicated with individual values in red dots. *p < 0.05, **p < 0.01 and ***p < 0.001 compared to siCont by Student’s *t*-test with Bonferroni multiple comparison correction.

The involvements of COX pathway in the regulation of DNA methylation have been previously reported. Treatment with celecoxib, a COX-2 specific inhibitor, suppressed DNA methylation in the promoter region of *ESR1* encoding the estrogen receptor α in rat colon tumor cells^34^. Similarly, aspirin, a non-selective NSAID, lowered DNA methylation in the promoter region of *CDH1* encoding cadherin 1 in human gastric mucosa cells^35^. Overexpression of prostaglandin E2 (PGE2), one of the products of COX enzyme, elevated expression of DNA methyltransferase 3α (DNMT3a), which is necessary for *de novo* DNA methylation^36^, and increased DNA methylation in the promotor regions of 13 genes in fetal lung fibroblast cells^37^. In addition, overexpression of COX-2 in hepatocytes downregulated expression of tet methylcytosine dioxygenase 1 (TET1), and induced promoter hypermethylation of 68 genes^38^. TET1 is a member of ten-eleven translocation (TET) family proteins that promote DNA demethylation through oxidation of 5-methylcytosine^39^. We observed that knockdown of COX-1, but not of COX-2, upregulated mRNA expressions of all three members of TET (*Tet1*, *Tet2*, and *Tet3*) (Fig. 7). We consistently observed that inhibition of COX-1, but not of COX-2, demethylated MeR2 (Fig. 6 and Supplementary Fig. S4). In contrast, knockdown of neither COX-1 nor COX-2 affected *Dnmt3a* mRNA expression (Fig. 7). These results suggest a role of TET in the COX-1-dependent suppression of MeR2-methylation in C2C12 cells, although additional molecules may also be involved in the suppression.

The promoter/enhancer-dependent transcription of *Mbnl1* in human and mouse remains largely unknown. In contrast, in *Drosophila*, two enhancers located in *Mbnl* intron 2 specifically drive *Mbnl* expression in embryonic somatic muscle and the nerve cord. These enhancers are enriched in consensus binding sites for Mef2 transcription factor^20^, which is necessary for the expression of MBNL in *Drosophila*^7^. Although the sequences of the *Drosophila Mbnl* enhancers are not conserved in mammals, we found that MeR2 has three Mef2 binding sites according to JASPAR (http://jaspar.genereg.net/). As DNA methylation interferes with bindings of transcription factors to enhancers and promoters^40,41^, methylation of MeR2 may inhibit the binding of Mef2 to suppress transcription of *Mbnl1*.

MBNL1 regulates a large subset of alternative splicing transition^8,42^. Genes involved in skeletal muscle development and function, including *Bin1*, *Clcn1*, *Ldb3*, *Serca1*, and *Tnnt3*, are aberrantly spliced in *Mbnl1*-knockout mice as well as in DM1 patients, in which MBNL1 is sequestrated to the nuclear RNA foci with abnormally expanded CUG-repeats^43–45^. These missplicing events lead to muscle weakness^45^ and myotonia^8^ in DM1 skeletal muscle, where splicing isoforms are shifted from adult forms to immature forms^46,47^. A previous study showed that 2-fold upregulation of MBNL1 protein by transduction of a recombinant adeno-associated viral vector reversed missplicing of *Clcn1*, *Ldb3*, *Serca1*, and *Tnnt3*, resulting in a significant reduction in myotonia in a DM1 mouse model^17^. We also reported that ~1.5-folds upregulation of MBNL1 protein by phenylbutazone, a non-selective NSAID, reversed missplicing of *Clcn1*, *Nfix*, and *Rpn2*, and improved muscle weakness in a DM1 mouse model^21^. These results suggest that upregulation of MBNL1 effectively ameliorates aberrant splicing in DM1 muscle cells, in which the MBNL1-availability is severely impaired. In the current study, we found that COX-inhibitions increased MBNL1 ~1.5-folds (Fig. 2 and Supplementary Fig. S1c), which is likely to sufficient to exert some beneficial effects in DM1 cells. In contrast, in normal C2C12 cells, we observed that knockdown of COX-1 did not obviously affect the splicing of *Clcn1* as well as expressions of myogenic regulatory factors, MyoD and Myogenin (Supplementary Fig. S5), suggesting that upregulation of MBNL1 by NSAIDs is unlikely to be toxic in normal cells, in which MBNL1 is abundantly available.

Enhancement of MBNL1 expression is one of the attractive therapeutic modalities for DM1^17–19^. Our study shows that nearly a half of the screened NSAIDs enhanced *Mbnl1* expression in myogenic cells, suggesting possible application of NSAIDs for DM1 patients. Indeed, phenylbutazone, a non-selective COX inhibitor, ameliorates muscle weakness and muscle pathology in DM1 mouse model^21^. The beneficial effects of NSAIDs on skeletal muscles are not limited to DM1. Chronic administration of NSAIDs, such as ibuprofen and acetaminophen, increases muscle volume and muscle strength in healthy aged adults^48^. Furthermore, several NSAIDs have been reported to prevent muscle damage and preserve the number and function of muscle cells in a mouse model of Duchenne muscular dystrophy^49,50^. NSAIDs have adverse effects on the gastrointestinal tract, the kidneys, and the cardiovascular system^51^. In addition, a recent study revealed that the long-term use of NSAIDs has an influence on the diversity of fecal microbiota^52^. These issues should be taken into account in clinical trials in the future. Although further analyses are required, we expect that COX-1-selective NSAIDs and possibly their derivatives will become one of therapeutic options for DM1 patients.

## Methods

### Classification of NSAIDs

NSAIDs were classified based on the relative inhibitory activities against COX-1 and COX-2. According to a previous report^53^, NSAIDs with the IC_50_ ratio (COX-2 IC_50_/COX-1 IC_50_) > 5 were classified as COX-1-selective inhibitors, and those with the IC_50_ ratio < 0.2 were classified as COX-2-selective inhibitors. Although the IC_50_ ratios of celecoxib and meloxicam were 0.70 and 0.37, respectively, these drugs were classified as COX-2-selective inhibitors according to the IC_80_ ratios^53^. In addition, celecoxib and meloxicam have been deemed as COX-2 inhibitors in clinical and biochemical studies^54,55^. We thus classified celecoxib and meloxicam as COX-2-selective inhibitors. The other NSAIDs lacking the IC_50_ ratio^53^ were classified as follows: tenoxicam, acemetacin and oxprozin were classified as COX-1-selective NSAIDs according to the previous reports^56–58^. Acetaminophen was classified as a COX-2-selective NSAID^59^, and the remaining NSAIDs were classified as non-selective NSAIDs^60–64^. The classification of the NSAIDs is summarized in Table 1 and IC_50_ values of NSAIDs are shown in Supplementary Table 1.

**Table 1:** Classification of NSAIDs according to COX-1 and COX-2 inhibitory activities.

COX selectivity	Drugs
COX-1-selective	ketoprofen, flurbiprofen, suprofen, tenoxicam, acemetacin, oxprozin
COX-2-selective	nimesulide, etoricoxib, rofecoxib, meloxicam, celecoxib, acetaminophen, etodolac
non-selective	piroxicam, naproxen, ibprofen, tolmetin, meclofenamate, aspirin, nabumetone, fenoprofen, flufenamate, diclofenac, isoxicam, indoprofen, mesalamine, fenbufen, azapropazone, phenylbutazone

### C2C12 cell culture and drug screening

C2C12 myoblasts were cultured in Dulbecco’s modified Eagle’s medium (DMEM) supplemented with 10% (v/v) fetal bovine serum (FBS) and incubated at 37 °C with 5% CO_2_. When the cells were grown to 50–60% confluency, 10 μM or 100 μM of each NSAID (Prestwick Chemical Library) was added. After 24 h, cells were harvested and total RNA was extracted as described below.

To examine the effect of naproxen (02190247, MP Biomedicals) on MBNL1 expression in C2C12 myotubes, C2C12 myoblasts were grown to 95% confluency in DMEM with 10% FBS, and then the medium was changed to DMEM with 2% horse serum to induce myotube differentiation. Naproxen was dissolved in dimethyl sulfoxide (DMSO) to make 100 and 400 mM solutions separately, and then added to the culture medium at final concentrations of 100 and 400 μM at the initiation of differentiation. DMSO (0.1%) was also added to the medium of control cells. After 5 days, gene expression of *Mbnl1* mRNA was quantified using real-time RT-PCR, and the amount of MBNL1 protein was estimated by Western blotting. All samples were analyzed in triplicates.

### Primary myoblast cell and KD3 myoblast cell culture

All experiments using mice were approved by the Animal Care and Use Committee of the Nagoya University and were performed in accordance with the relevant guidelines. The pectoralis major, gastrocnemius, and quadriceps were removed from ten neonatal mice at 9 days of age. Skeletal muscles were minced in sterile PBS under the microscope with sterile forceps. Following addition of an appropriate amount of trypsin, the minced muscles were incubated at 37 °C for 2 h to so that the muscles were fully digested to a slurry. Then two volumes of growth medium [DMEM with 20% HS, 10% FBS, 2% penicillin, 1% glutamine and 20 U/ml interferon γ (PeproTech)] were added, and the slurry was filtered through 440- and 70-μm nylon mesh (Corning Costar) to remove large pieces of tissue. After centrifugation of the slurry at 300 × *g* for 10 min, the pellet was suspended in the growth medium and centrifuged again at 8 × *g* for 1 min to precipitate blood cells. Then, the supernatant was filtered through 35-μm nylon mesh and the cells were plated in 2 ml of the growth medium in a 60-mm collagen-coated culture dish.

KD3 myoblasts were kindly provided by Dr. Naohiro Hashimoto at the National Center for Geriatrics and Gerontology, Japan^65^. KD3 myoblasts were cultured in DMEM supplemented with 20% (v/v) FBS, 2% Ultroser G (Biosepra, PALL) and incubated at 37 °C with 5% CO_2_. To differentiate KD3 cells, the cells were grown to 95–100% confluency, medium were changed to DMEM with 2% (v/v) FBS, 5 μg/ml holo-transferrin (bovine) and 10 μg/ml insulin (Sigma). All samples were analyzed in triplicates. The medium was replaced every two days.

### MyoD-DM1-iPSCs cell culture

DM1 patient-derived iPSCs (MyoD-DM1-iPSCs) were cultured essentially as described elsewhere with minor modifications^32,33^. Briefly, MyoD-DM1-iPSCs were cultured on a plate coated with iMatrix-511 (Takara, 892011) in StemFit (Takara, AK02N) containing 125 ng/ml puromycin and 10 μM Y-27632 (Wako, 030-24021). Cells were passaged every 6 or 7 days using Accutase (Gibco, A1110501) and seeded on an iMatrix-511-coated six-well plate at the density of 15,000 cells/well.

To differentiate MyoD-DM1-iPSCs into myotubes, the cells were detached with Accutase and seeded on a Matrigel (Corning)-coated six-well plate in StemFit with 10 μM Y-27632 at the density of 200,000 cells/well. At 24 h after differentiation (differentiation day 1), the medium was changed to PECM (Primate ES Cell Medium, Reprocell) containing 10 μM Y-27632. At 24 additional hours (differentiation day 2), the medium was changed to PECM containing 1 μg/ml Dox (dxycycline). On differentiation day 3, MyoD-DM1-iPSCs were treated with Accutase and changed to new a Matrigel-coated plate in αMEM (Nacalai Tesque) with 5% KSR (KnockOut™ Serum Replacement, Gibco), 200 μM 2-ME (2-mercaptoethanol), and 1 μg/ml Dox. The medium was changed every other day with fresh αMEM + 5% KSR + 200 μM 2-ME + 1 μg/ml Dox.

### Splicing analysis and gene expression analysis

Total RNA was extracted by RNeasy Mini Kit (Qiagen) according to the manufacturer’s instructions, and reverse-transcribed to cDNA using random hexamer primers (ThermoFisher) and ReverTraAce (Toyobo). PCR amplifications were performed using GoTaq (Promega) for 30 cycles. The primer sequences used for RT-PCR are shown in Supplementary Table S2. The intensities of RT-PCR-amplified spliced products were quantified with the ImageJ program (http://imagej.nih.gov/ij/). We then estimated the ratio of exon inclusion by dividing the signal intensity of the upper band by the sum of signal intensities of two bands. All samples were analyzed in triplicates.

Real-time RT-PCR was performed with the LightCycler 480 (Roche Applied Science) using the TB Green Premix ExTaq II (Takara Bio). Gene expression levels were normalized by that of glyceraldehyde-3-phosphate dehydrogenase (*Gapdh*). PCR primers are shown in Supplementary Table S2. All real-time RT-PCR experiments were performed in triplicate. All samples were analyzed in triplicates.

### Western blot analysis

Cells were washed with cold PBS twice, and were lysed with PLC buffer [50 mM HEPES pH 7.0, 150 mM NaCl, 10% (vol/vol) glycerol, 1% (vol/vol) TritonX-100, 1.5 mM MgCl_2_, 1 mM EGTA, 100 mM NaF, 10 mM NaPPi (sodium pyrophosphate)] with protease inhibitors (1 μg/μl aprotinin, 1 μg/μl leupeptin, and 1 mM PMSF). Then, the lysates were gently sonicated twice for 1 min using Handy Sonic (Tomy Seiko) at intensity levels 3 to 4, and were cleared with centrifugation for 15 min at 15,000 × *g* at 4 °C. The total protein concentrations of the lysates were measured using Pierce 660 nm Protein Assay Reagent. Cell lysates were boiled for 5 min in 2 × Laemmli buffer, separated on a 7.5% or 10% SDS-polyacrylamide gel, and transferred to a polyvinylidene fluoride membrane (Immobilon-P, Millipore). Then, the membrane was blocked for 1 h at room temperature in TBS-T with 5% skim milk. After washing with Tris-buffered saline containing 0.05% Tween 20 (TBS-T), the membrane was incubated overnight at 4 °C with primary antibodies listed in Supplementary Table S3. Next, the membrane was washed with TBS-T and incubated with secondary goat anti-mouse IgG (1:3000, LNA931V/AG, GE Healthcare) or anti-rabbit IgG (1:3000, LNA934V/AE, GE Healthcare) antibody conjugated to horseradish peroxidase (HRP) for 1 h at room temperature. The bound antibodies were detected with ECL Western blotting detection reagents (GE Healthcare), and the signal intensities were quantified with the ImageJ program (http://imagej.nih.gov/ij/). All samples were analyzed at least in triplicates.

### Knockdown of COX-1 and COX-2 in myogenic cells

siRNA duplexes against COX-1 and COX-2 were synthesized by Sigma-Aldrich. The siRNA sequences for mouse COX-1 and COX-2 were: siCOX1-a, 5′-GGCUUAAAACUUUAUAUUA-3′; siCOX1-b, 5′-GCAUCGCCAUGGAAUUUAA-3′; siCOX2-a, 5′- GGAGCUUCCUGAUUCAAAA-3′; and siCOX2-b, 5′- GGAUUUGACCAGUAUAAGUUU-3′. The siRNA sequences for human COX-1 and COX-2 were: siCOX1-c, 5′-GGAGGAAGUUCAUACCUGA-3′; siCOX1-d, 5′-GCAUUGCCAUGGAGUUCAA-3′; siCOX2-c, 5′- GGAAUUUUUGACAAGAAUA-3′; and siCOX2-d, 5′- GGACUUAUGGGUAAUGUUA-3′. The control siRNA was AllStar Negative Control siRNA (1027281, Qiagen). siRNAs were introduced into cultured cells using Lipofectamine RNAiMax (ThermoFisher) according to the manufacturer’s recommendations.

For knocking down of COX-1 or COX-2 in undifferentiated C2C12 myoblasts, primary myoblast cells and undifferentiated KD3 myoblasts, siRNAs were introduced when cells were grown to 50–60% confluency. For knocking down of COX-1 or COX-2 in differentiated C2C12 myotubes and differentiated KD3 myotubes, cells were first grown to 95% confluency and then medium was changed to differentiation medium and the cells were transfected with siRNAs. For knocking down of COX-1 or COX-2 in MyoD-DM1-iPSCs, siRNAs were introduced at 24 h after replating (Fig. 3a). After incubation for 2 additional days, new medium was added and siRNAs were introduced again. Total RNA or protein lysates were extracted at the indicated time points. mRNA expression was quantified by real-time RT-PCR and protein expression was analyzed by Western blotting as stated above.

### Immunofluorescence staining of MyoD-DM1-iPSCs

Myotubes differentiated from MyoD-DM1-iPSCs were immunostained essentially as described elsewhere with minor modifications^33^. Briefly, MyoD-DM1-iPSCs were fixed with 2% PFA/PBS on 5 days after replating. Then, cells were blocked with Blocking One (Nacalai Tesque) for 4 h at 4 °C and subsequently incubated at 4 °C overnight with rabbit anti-myosin heavy chain (anti-MHC) antibody (1:50, sc-20641, Santa Cruz Biotechnology), which was diluted in 10% Blocking One/PBST. Cells were washed in PBS and incubated with anti-rabbit FITC (1:100, Vector Laboratories) diluted in 10% Blocking One/PBST for 1 h at room temperature and washed with PBST. DAPI was used to counterstain the nuclei.

### Generation of the MeR2-knockout (MeR2-KO) C2C12 cell line by CRISPR/Cas9 system

To generate the MeR2-knockout C2C12 cell line, we used the CRISPR/Cas9 system in conjunction with dual sgRNAs. The online CRISPRdirect webtool (https://crispr.dbcls.jp/) was used to design sgRNAs targeting the MeR2 region (Fig. 4a). The CRISPR/Cas9 target sequences, which were comprised of a 20-bp target sequence and a 3-bp protospacer-adjacent motif (PAM) sequence (underlined), were: sgRNA-1, 5′-ATACACACTATCACTCAATCTGG-3′; and sgRNA-2, 5′-GGAGAAAGCGATTCCATTGCAGG-3′. The templates for *in vitro* transcription of sgRNA were amplified by PCR using the pX330 vector (Addgene) as template DNA with the following primers: forward primer, 5′-TAATACGACTCACTATAGGG-[20-bp sgRNA target sequence]-GTTTTAGAGCTAGAAATA-3′, and reverse primer; 5′-AAAAGCACCGACTCGGTGCC-3′. The underlined sequence in the forward primer indicates the T7 promoter region. The segment of tracer RNA was obtained by PCR using the pX330 vector as a template. The amplified PCR fragments were purified with AMPure XP beads (Beckman Coulter), and was *in vitro* transcribed using RiboMAX Large Scale RNA Production Systems-T7 (Promega) according to the manufacturer’s instructions. The sgRNA was purified with Quick-RNA MicroPrep Kit (ZYMO RESEARCH).

C2C12 cells were transfected with sgRNA-1, sgRNA-2, and Cas9 enzyme (Integrated DNA Technologies) using Lipofectamine RNAiMax (ThermoFisher) according to the manufacturer’s recommendations. Two days after transfection, cells were seeded at 0.5 cells/well and 0.2 cells/well in 96-well plates. After 10 days, all single cell clones were screened with PCR amplification of the genomic segment harboring MeR2 region, using a pair of primers, 5′-ATGGCTTCAGGGTATTCAGCC-3′ and 5′-GGCGAGGGATGAACAAAAGC-3′. The expected size of PCR amplicons of MeR2-KO clones was around 165 bp, while that of the MeR2-retained wild-type clones was around 1900 bp. Following the PCR amplification, the amplicons were cloned into pGEM-T vector (Promega) and sequenced to determine the deleted segment of the MeR2 (Fig. 4b). We obtained one single cell clone, in which MeR2 was deleted in both alleles (MeR2-KO C2C12). Another single cell clone retaining MeR2 on both alleles was used as a WT control (WT C2C12).

### Transfection and luciferase assays

To make the luciferase vector harboring the MeR2 enhancer (pGL3P-MeR2), the mouse genomic region from positions 60,330,708 to 60,331,205 on chromosome 3 according to NC_000069.6 (GRCm37/mm9) was PCR-amplified with the following primers; 5′-CATGGTACCCAGTAATCTGAGTCCTGCTGTAGTAA-3′ and 5′-GATGCTAGCGAAGACATCTACCTATGCTAAAAGCA-3′, where KpnI and NheI restriction sites are underlined, respectively. Then the amplified DNA fragment was cloned into the pGL3-Promotor Vector (pGL3P, Promega) at KpnI and NheI restriction sites. The pGL3P-MeR2 and pGL3P were introduced into XL-10 gold competent cells (Agilent) and were propagated. To measure the transcriptional activity of MeR2 enhancer, C2C12 myoblasts were transiently transfected with pGL3P-MeR2 or pGL3P using Lipofectamine 3000 (ThermoFisher) according to the manufacturer’s recommendations, and were incubated for 24 h. Then, 100 μM of each NSAID was added to the culture medium and cells were cultured for an additional 24 h. Alternatively, siRNAs were introduced into cells with Lipofectamin RNAiMax (ThermoFisher), and cells were cultured for an additional 48 h. The luciferase assay was performed using the Dual Luciferase Reporter Assay System kit (Promega). To normalize for the transfection efficiency and the cell number, pRL-CMV Renilla Luciferase Reporter Vector was cotransfected, and the firefly luciferase activities were normalized to the Renilla luciferase activity. All samples were analyzed at least in triplicates.

### DNA extraction and CpG methylation analysis by bisulfite-sequencing PCR

Bisulfite-sequencing PCR (BSP) was performed as we previously reported with minor modifications^21^. Briefly, genomic DNA was extracted from differentiating C2C12 myoblasts on differentiation day 3 using QIAamp DNA Mini Kit (Qiagen). Then, the DNA was treated with the MethylEasy Xceed Rapid DNA Bisulfite Modification Kit (Human Genetic Signatures) according to the manufacturer’s instructions to convert non-methylated cytidines to uridines. A pair of BSP primers (Supplementary Table S2) were used to amplify the MeR2 region of *Mbnl1*. The PCR products were run on an agarose gel, and the excised bands were purified with the Wizard SV Gel and PCR Clean-Up System (Promega). The PCR products were subcloned into the TA cloning vector (pGEM-T Easy Vector, Promega). To identify the methylated CpG dinucleotides in the MeR2 region, 14 independent clones for each sample were sequenced with the reverse BSP primer at Eurofins Genomics (Japan). The methylation pattern was analyzed with the QUMA (Quantitative Tool for Methylation Analysis) software (http://quma.cdb.riken.jp/) using default parameters^66^.

## Supplementary information

Supplementary file.
