# Supplementary material for: Inhibition of cyclooxygenase-1 by nonsteroidal anti-inflammatory drugs demethylates MeR2 enhancer and promotes Mbnl1 transcription in myogenic cells
Source: Sci Rep. 2020 Feb 13;10:2558. doi: 10.1038/s41598-020-59517-y (PMC7018979; doi:10.1038/s41598-020-59517-y)
Supplement: Supplementary file 1 — Supplementary file. [file 41598_2020_59517_MOESM1_ESM.docx]

**Inhibition of cyclooxygenase-1 by nonsteroidal anti-inflammatory drugs demethylates MeR2 enhancer and promotes *Mbnl1* transcription in myogenic cells**

**Kun Huang^1^, Akio Masuda^1,*^, Guiying Chen^1^, Samira Bushra^1^, Masayoshi Kamon^2^, Toshiyuki Araki^2^, Masanobu Kinoshita^3^, Bisei Ohkawara^1^, Mikako Ito^1^, Kinji Ohno^1^**

^1^Division of Neurogenetics, Center for Neurological Diseases and Cancer, Nagoya University Graduate School of Medicine, Nagoya, Aichi, Japan

^2^Department of Peripheral Nervous System Research, National Institute of Neuroscience, National Center of Neurology and Psychiatry, Kodaira, Tokyo, Japan

^3^Mumin Clinic Omiyakita, Saitama, Saitama, Japan

***Correspondence:** Akio Masuda, MD, PhD, Division of Neurogenetics, Center for Neurological Diseases and Cancer, Nagoya University Graduate School of Medicine, 65 Tsurumai, Showa-ku, Nagoya 466-8550, Japan. e-mail: amasuda@med.nagoya-u.ac.jp

**Supplementary information includes:**

**Supplementary Table S1-S3.**

**Supplementary Figures S1-S7.**

**Supplementary Table S1. IC_50_ of NSAIDs and the ratio of COX-2 IC_50_ to COX-1 IC_50_**

| NSAIDs | COX-1 IC_50_^*^ (μM) | COX-2 IC_50_  (μM) | IC_50_ ratio  (COX-2 IC_50_/COX-1 IC_50_) |
| --- | --- | --- | --- |
| Rofecoxib | 63 | 0.31 | 0.00 |
| Etoricoxib | - | - | - |
| Diclofenac | 0.075 | 0.02 | 0.27 |
| Flufenamic | 3 | 9.3 | 3.10 |
| Fenoprofen | 3.4 | 5.9 | 1.74 |
| Celecoxib | 1.2 | 0.34 | 0.28 |
| Isoxicam | - | - | - |
| Aspirin | 1.7 | 7.5 | 4.41 |
| Nabumetone | 460 | 290 | 0.63 |
| Acetaminphen | - | - | - |
| Meclofenamic | 0.22 | 0.2 | 0.91 |
| Meloxicam | 5.7 | 0.23 | 0.04 |
| Tolmetin | 0.35 | 1.3 | 3.71 |
| Indoprofen | - | - | - |
| Suprofen | 1.1 | 8.3 | 7.55 |
| Flubiprofen | 0.075 | 0.77 | 10.27 |
| Nimesulide | 10 | 0.39 | 0.04 |
| Ibuprofen | 7.6 | 20 | 2.63 |
| Tenoxicam | - | - | - |
| Mesalamine | - | - | - |
| Etodolac | 12 | 0.2 | 0.02 |
| Acemetacin | - | - | - |
| Febufen | - | - | - |
| Azapropazone | - | - | - |
| Oxprozin | - | - | - |
| Naproxen | 9.3 | 35 | 3.76 |
| Piroxicam | 2.4 | 7.9 | 3.29 |
| Ketoprofen | 0.047 | 0.24 | 5.11 |
| Phenylbutazone | - | - | - |

* IC50: IC50 is the concentration of a drug that is required for 50% inhibition *in vitro*.

**Supplementary Table S2. Primer sequences for real-time RT-PCR and bisulfite sequencing**

| **Gene** | **Forward primer (5'-3')** | **Reverse primer (5'-3')** |
| --- | --- | --- |
| Primers for mouse genes | | |
| *Gapdh* | ACCCCTTCATTGACCTCAAC | TCCCGTTGATGACAAGCTTC |
| *Mbnl1* | GCGTGGCAATTGCAACAG | GCATCTCCCCTTGATGTAATCC |
| *Ptgs1* (COX-1) | TCCTGGCTTTACCAAGGCCT | GGTACACCTCTCCGTCCAGC |
| *Ptgs2* (COX-2) | TGGGTGTGAAGGGAAATAAGG | CATCATATTTGAGCCTTGGGG |
| *Tet1* | AGCGCATCAGAAAACGGCTA | GACTTGAATGTTCTGTTCTGGCC |
| *Tet2* | GAGTCACTGCATGTTTGGACTTCTC | GCTCCGACTTCTCGATTGTCTTC |
| *Tet3* | GAGGAACATCCATCCTTTGCTCC | CTGCTCCAGTTCTGCCATAGG |
| *MeR2* | GGAGGGTTTGTATGATTTTGGGATTTG | CCTTCTCTTAACTCAAAAACCCACTC |
| *Clcn1* | GTCCTCAGCAAGTTTATGTCC | GAATCCTCGCCAGTAATTCC |
| Primers for human genes | | |
| *GAPDH* | GTCTCCTCTGACTTCAACAGCG | CCACCCTGTTGCTGTAGCCAA |
| *MBNL1* | CTAGCCAATGCCATGATGCCTG | CCTGTAACCAACATTGGTGCAGTC |
| *PTGS1*  *(COX-1)* | CTTCTGGCAAGATGGGTCCTG | CAACACAGGCGCCTCTTCTAC |
| *PTGS2*  *(COX-2)* | GGAGAGATGTATCCTCCCACAGTC | GTCTGGAACAACTGCTCTACACC |

**Supplementary Table S3. Antibodies and dilutions for Western blotting**

| Antigen | Manufacturer | Species | Catalog No. | Dilution |
| --- | --- | --- | --- | --- |
| COX-1 | Abcam | Rabbit | ab109025 | 1:1000 |
| COX-2 | Cell Signaling  Technology | Rabbit | #4842 | 1:500 |
| MBNL1 | Santa Cruz | Mouse | sc-136165 | 1:200 |
| GAPDH | Sigma-Aldrich | Rabbit | G9545 | 1:1000 |
| MyoD | Santa Cruz | Rabbit | sc-304 | 1:200 |
| Myogenin | Santa Cruz | Rabbit | sc-576 | 1:200 |

**Supplementary figures**

**
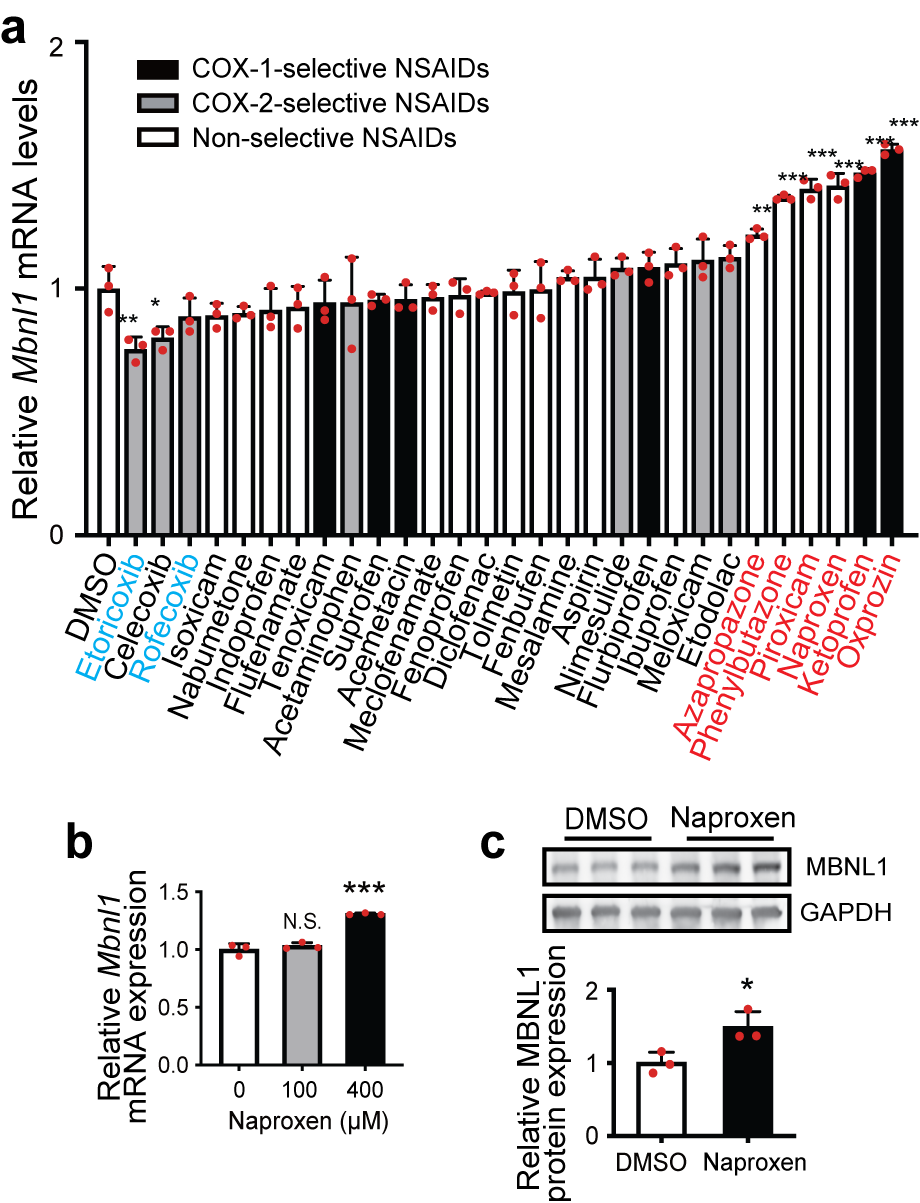
**

**Supplementary Figure S1.** **Screening of 29 NSAIDs on *Mbnl1* mRNA expression at 10 µM. (a)** Undifferentiated C2C12 cells were cultured with 10 μM each of NSAIDs for 24 h. Expression levels of *Mbnl1* and *Gapdh* were quantified by real-time RT-PCR. Expression level of *Mbnl1* was normalized to that of *Gapdh*, and also to control cells with only DMSO. Drugs that upregulated *Mbnl1* more than 1.4-folds and decreased *Mbnl1* less than 0.7-folds at 100 µM in Fig. 1 are indicated in red and blue letters, respectively. Mean and SD (*n* = 3 culture dishes) are shown with individual values in red dots. **p* < 0.05, ***p* < 0.01, and ****p* < 0.001 compared to DMSO alone by Student's *t*-test with Bonferroni multiple comparison correction. Fig. 1 used 100 µM NSAIDs, whereas the current experiment used 10 µM NSAIDs. **(b)** Real-time RT-PCR to estimate expression of *Mbnl1* in differentiated C2C12 myotubes. Cells were added with 0, 100, and 400 µM naproxen, and differentiation was initiated simultaneously. Real-time RT-PCR analysis was performed on differentiation day 5. Expression levels of *Mbnl1* are normalized to that of *Gapdh*, and also to that of cells treated with 0 µM naproxen. The mean and SD (*n* = 3 dishes) are indicated with individual values in red dots. ****p* < 0.001 and N.S., not significant, compared to 0 µM naproxen by Student's *t*-test with Bonferroni multiple comparison correction. **(c)** Western blotting analysis of MBNL1 in differentiated C2C12 myotubes. Cells were treated with 400 μM naproxen for 5 days during myogenic differentiation. Mean and SD (*n* = 3 culture dishes) are indicated with individual values in red dots. **p* < 0.05 by Student's *t*-test.


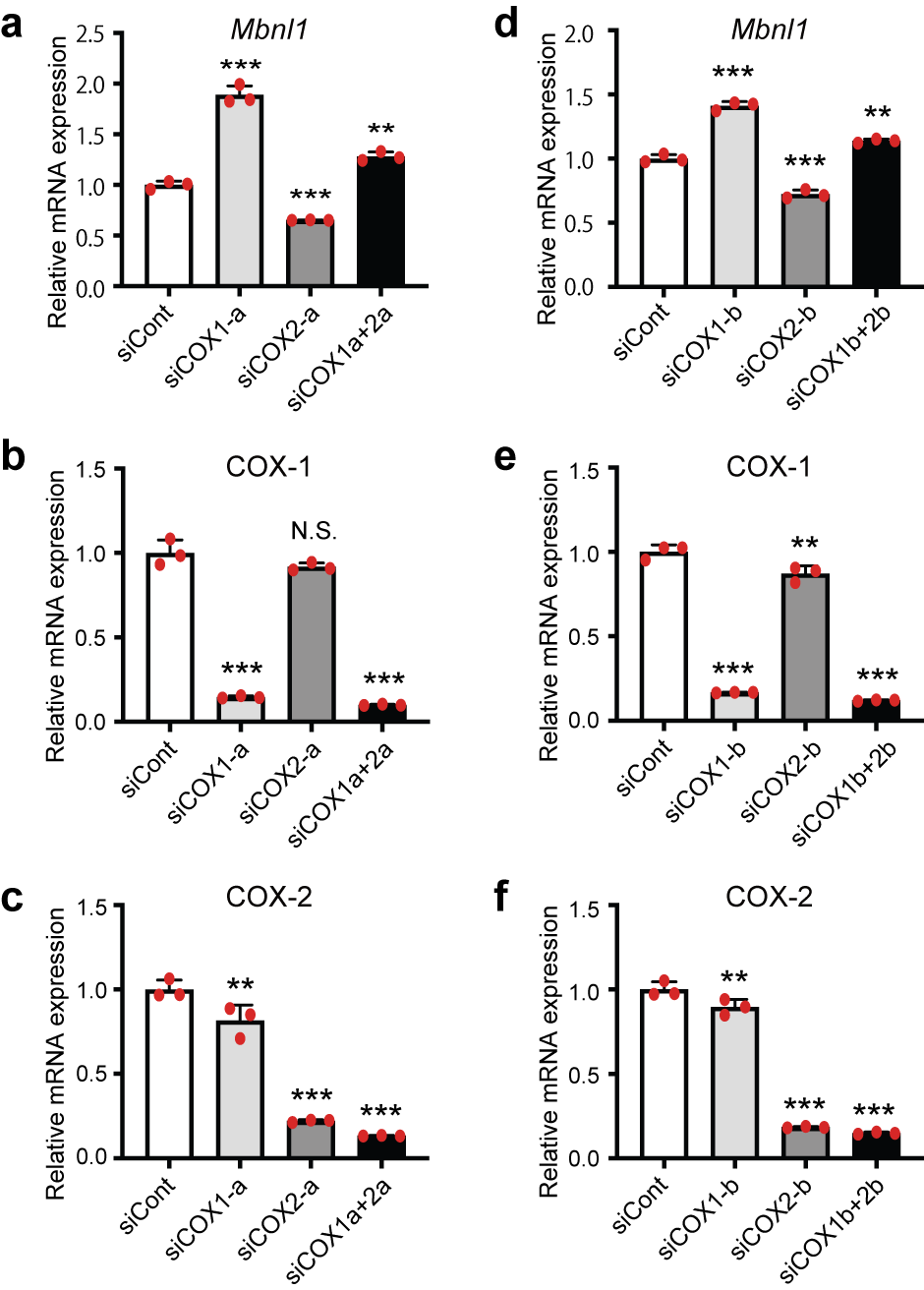


**Supplementary Figure S2. The effect of knockdown of COX-1 and COX-2 on *Mbnl1* mRNA expression in primary myoblast cells.** Real-time RT-PCR analysis was performed using primary myoblasts. The cells were treated with siRNA against COX-1 (siCOX1-a or siCOX1-b), COX-2 (siCOX2-a or siCOX2-b), both COX-1 and COX-2 (siCOX1a+2a or siCOX1b+2b), or control siRNA (siCont). The cells were examined 2 days after the transfection. Expression levels of *Mbnl1* mRNA **(a, d)**, *Ptgs1* (COX-1) mRNA **(b, e)**, and *Ptgs2* (COX-2) mRNA **(c, f)** are normalized to that of *Gapdh* and also to siCont-treated cells. Mean and SD (*n* = 3 culture dishes) are indicated with individual values in red dots. ***p* < 0.01, ****p* < 0.001, and N.S., not significant by Student's *t*-test with Bonferroni multiple comparison correction. In contrast to Fig. 2, in which C2C12 cells was analyzed, primary myoblast cells were used.


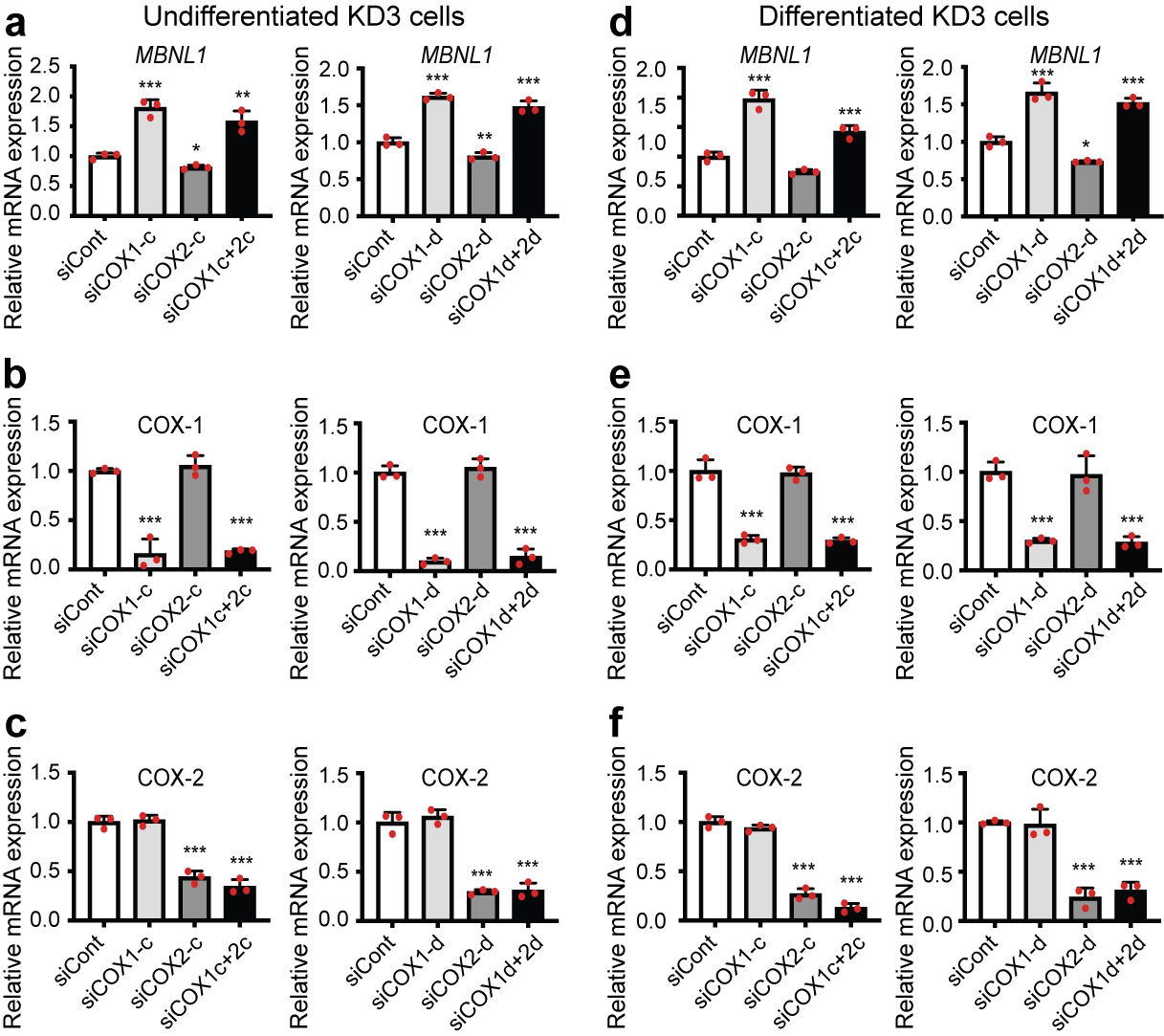


**Supplementary Figure S3. The effect of knockdown of COX-1 and COX-2 on *MBNL1* mRNA expression in KD3 cells.** Real-time RT-PCR analysis was performed using undifferentiated KD3 cells **(a, b, c)** or KD3 cells differentiated for 5 days **(d, e, f)**. The cells were treated with siRNA against COX-1 (siCOX1-c or siCOX1-d), COX-2 (siCOX2-c or siCOX2-d), both COX-1 and COX-2 (siCOX1c+2c or siCOX1d+2d), or control siRNA (siCont). Undifferentiated cells were examined on 2 days after the transfection. Differentiated cells were examined on differentiation day 5. Expression levels of *MBNL1* mRNA **(a, d)**, *PTGS1* (COX-1) mRNA **(b, e)**, and *PTGS2* (COX-2) mRNA **(c, f)** are normalized to that of *GAPDH* and also to siCont-treated cells. Mean and SD (*n* = 3 culture dishes) are indicated with individual values in red dots. **p* < 0.05, ***p* < 0.01 and ****p* < 0.001 compared to siCont by Student's *t*-test with Bonferroni multiple comparison correction. Fig. 2 used C2C12 mouse myogenic cells, whereas the current experiment used KD3 human myogenic cells.


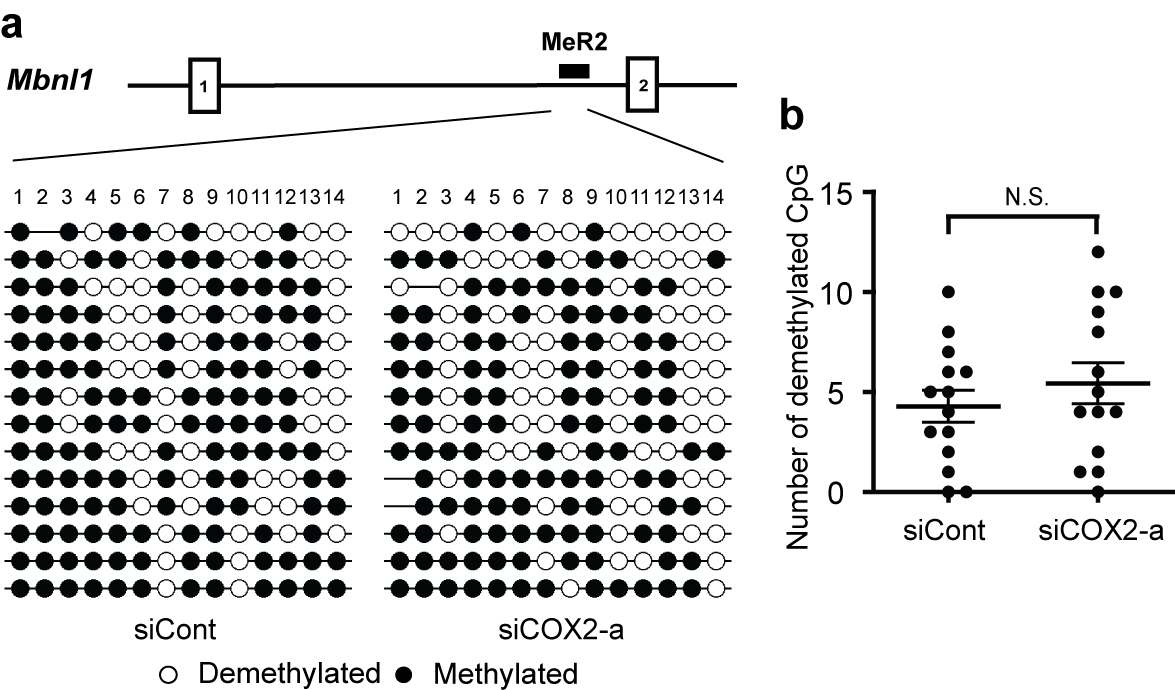


**Supplementary Figure S4. The effects of knockdown of COX-2 on methylation of CpG dinucleotides in MeR2 enhancer region*.*** C2C12 cells were transfected with siCOX2-a and DNA was extracted on differentiation day 3. Then the samples were treated with bisulfite sodium and cloned into the TA cloning vector for sequencing. For methylation analysis, 14 independent clones were sequenced for each group, and methylations of 14 CpG dinucleotides in MeR2 were analyzed. **(a)** Methylation analysis of MeR2. **(b)** The number of demethylated CpG in MeR2 was quantified individually at positions 1 to 14. The mean and SEM are indicated. N, S., not significant by paired Student’s *t*-test. In contrast to Fig. 4, in which siRNA against COX-1 was used, siCOX2-a was used to knock down COX-2.

**
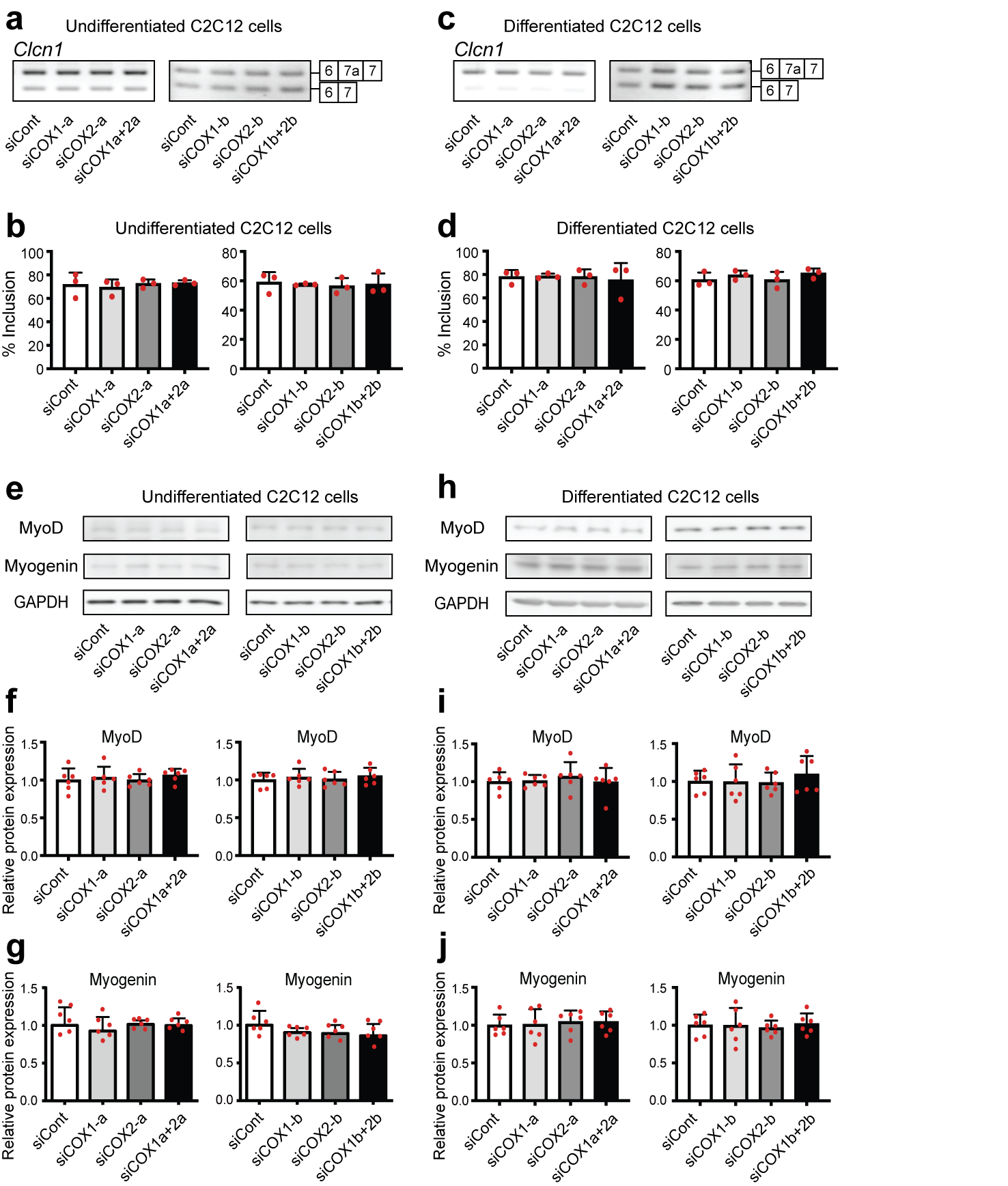
**

**Supplementary Figure S5. The effects of knockdown of COX-1 or COX-2 on splicing of *Clcn1* and expressions of MyoD and Myogenin in C2C12 cells.** Undifferentiated C2C12 cells or C2C12 cells in myogenic differentiation medium on day 0 were transfected with siRNA against COX-1 (siCOX1-a or siCOX1-b), COX-2 (siCOX2-a or siCOX2-b), both COX-1 and COX-2 (siCOX1a+2a or siCOX1b+2b), or control siRNA (siCont). Splicing of *Clcn1* exon 7a was analyzed by RT-PCR. Protein expression levels of MyoD and Myogenin were analyzed by Western blotting. **(a, b, e, f, g)** Undifferentiated C2C12 cells were examined 2 days after the transfection. **(c, d, h, i, j)** C2C12 cells in differentiation medium were examined on day 5. **(a, c)** RT-PCR analysis of splicing of *Clcn1* exon 7a. **(b, d)** The ratio of inclusion of *Clcn1* exon 7a was calculated as explained in Methods. Mean and SD (*n* = 3 culture dishes) are indicated with individual values in red dots. **(e, h)** Western blotting analysis of MyoD, Myogenin, and GAPDH. The images of MyoD and Myogenin were acquired with the same exposure time (30 sec). The expression of MyoD protein **(f, i)** and Myogenin protein **(g, j)** were normalized to that of GAPDH and also to siCont-treated cells. Mean and SD (*n* = 6 culture dishes) are indicated with individual values in red dots.


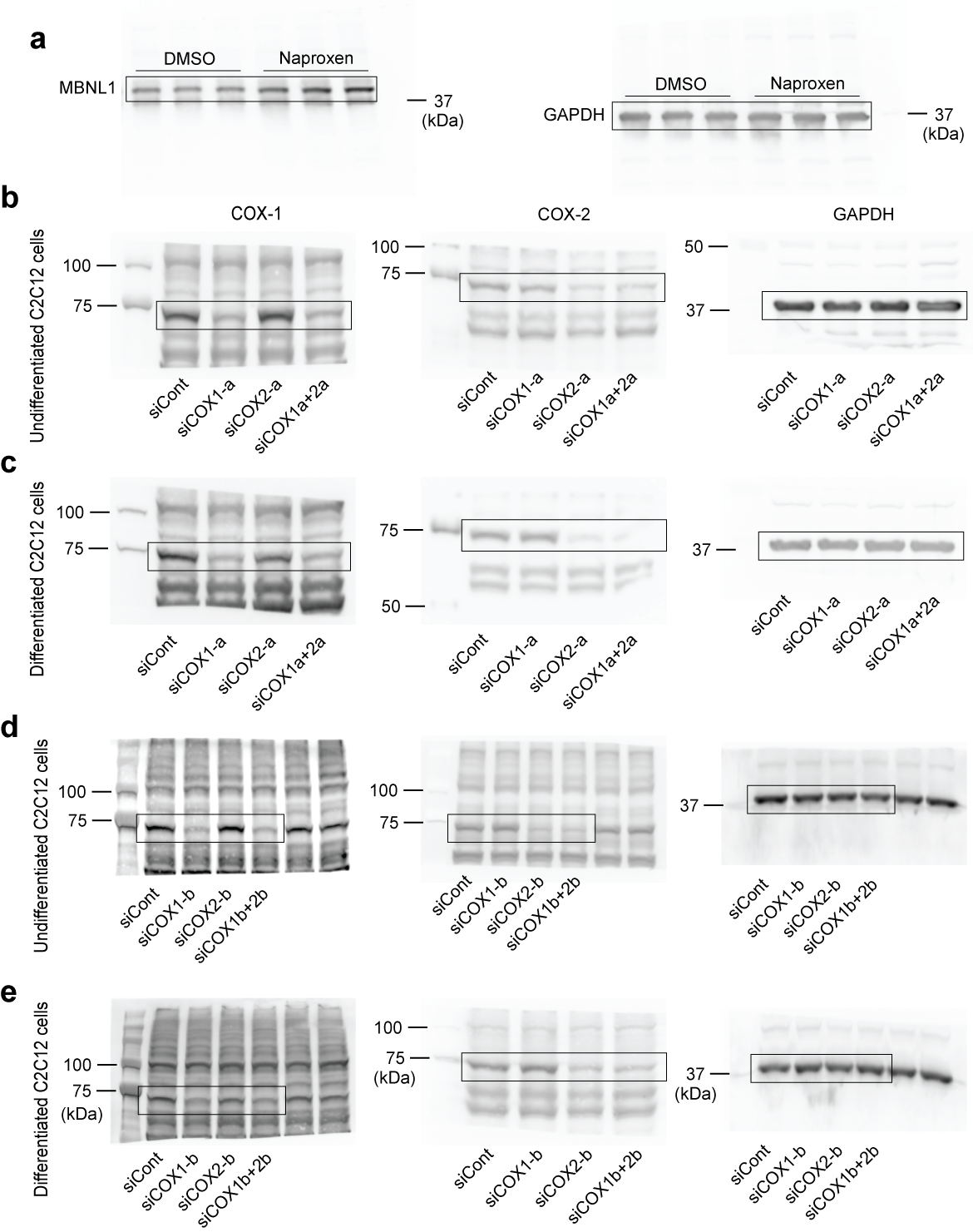


**Supplementary Figure S6. Full length images of the cropped gels.** Full length images of the cropped gels presented in Supplementary Fig. S1c **(a)**, Fig. 2a **(b, c)** and Fig. 2b **(d, e)**.


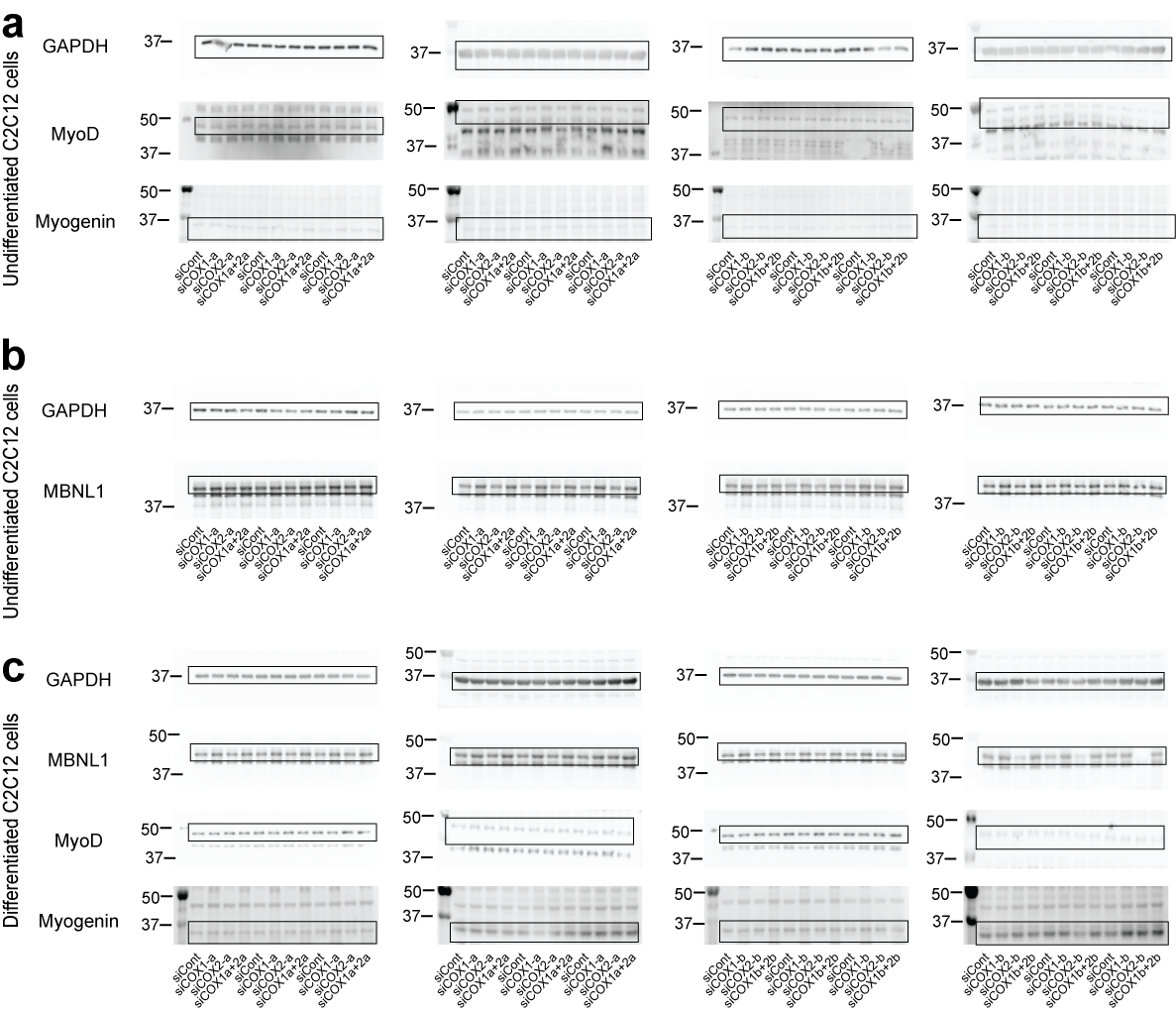


**Supplementary Figure S7. Full length images of the cropped gels.** Full length images of the cropped gels presented in Supplementary Fig. S5e **(a)**, Fig. 2g, h **(b),** Fig. 2g, h and Supplementary Fig. S5h **(c)**.
